# Mast Cell Deficiency in Mice Attenuates Insulin Phenolic Preservative-Induced Inflammation

**DOI:** 10.3390/biomedicines11082258

**Published:** 2023-08-12

**Authors:** Shereen Kesserwan, Marianna Sadagurski, Li Mao, Ulrike Klueh

**Affiliations:** Integrative Biosciences Center (IBio), Wayne State University, Detroit, MI 48202, USA; shereenkesserwan@gmail.com (S.K.); sadagurski@wayne.edu (M.S.);

**Keywords:** mast cells, inflammation, diabetes, automated insulin delivery, insulin infusion, insulin preservatives

## Abstract

One major obstacle that limits the lifespan of insulin infusion pumps is surmounting the tissue site reaction at the device implantation site. All commercial insulin formulations contain insulin phenolic preservatives (IPPs) designed to ensure insulin protein stability and prolong shelf-life. However, our laboratory demonstrated that these preservatives are cytotoxic and induce inflammation. Mature mast cells (MCs) reside in cutaneous tissue and are one of the first responders to an epidermal breach. Upon activation, MCs release proinflammatory and immunomodulatory prepacked mediators that exacerbate these inflammatory reactions. Thus, we hypothesized that once the epidermis is breached, cutaneous MCs are triggered inciting the inflammatory response to IPP-induced inflammation. This hypothesis was pursued utilizing our modified in vivo mouse air pouch model, including a c-*kit* dependent (*C57BL/6J-kit^W-sh/W-sh^*) and a c-*kit* independent (*Cpa3-Cre*; *Mcl-1^fl/fl^*) MC-deficient mouse model. Leukocytes were quantified in the mouse air pouch lavage fluid following flow cytometry analysis for IPP infusion under three different states, insulin-containing phenolic preservatives (Humalog^®^), insulin preservatives alone, and normal saline as a control. The air pouch wall was assessed using histopathological evaluations. Flow cytometry analysis demonstrated a statistically significant difference in inflammatory cell recruitment for both MC-deficient mouse models when compared to the control strain including infused control saline. Significantly less inflammation was observed at the site of infusion for the MC-deficient strains compared to the control strain. Overall, concordant results were obtained in both mouse types, *C57Bl6-kit^W-sh/W-sh^* and *Cpa3-Cre*; *Mcl-1^fl/fl^*. These findings in multiple model systems support the conclusion that MCs have important or possible unique roles in IPP-induced inflammation.

## 1. Introduction

The approved usage duration for commercial continuous-glucose-monitoring systems is 10+ days, whereas subcutaneous insulin infusion is 3+ limiting the possibility of an automated insulin-delivery system [1]. Several hypotheses have been proposed regarding the limited insulin infusion set functional lifespan, including inflammation induced by the material–tissue interface [2,3,4], insulin aggregate formation [5,6], or the toxicity of insulin phenolic preservatives (IPPs), such as phenol and m-cresol [7,8,9,10]. Specifically, our laboratory reported that these IPPs lead to unwanted cell and tissue toxicity [6,7,10], whereas Cromolyn sodium, an MC membrane stabilizer, significantly minimized IPP-induced inflammation [11]. These Cromolyn studies targeting MCs highlighted the potential to improve on automated insulin-delivery systems by mitigating the inflammatory response. Nonetheless, it is known that Cromolyn also affects various other cell functions, including neutrophils [12,13], indicating that results obtained might not be solely attributed to MCs.

Mature mast cells (MCs) reside in cutaneous tissue and are one of the first responders to a skin breach and are contributors in orchestrating the inflammatory response [14,15,16]. Once activated, MC granules release preformed mediators inducing an abundance of pro-inflammatory molecules, such as cytokines, chemokines, proteases, and lipid mediators [13,17]. Thus, we hypothesized that subcutaneous device implantation triggers MC activation and initiates the inflammatory response augmented by IPP delivery. This was investigated utilizing an in vivo mouse air pouch model [6,7,8,18], including a c-kit-dependent (*C57BL/6J-kitW-sh/^W-sh^*) and a *c-kit*-independent (*Cpa3-Cre*; *Mcl-1^fl/fl^*) MC-deficient mouse model. Since *c-kit* is a crucial stem cell factor receptor on mature MCs, with a key role in MC development, survival, and function [18,19,20], a mutation in this receptor leads to MC deficiency [21]. However, one limitation when using mice with a c-kit mutation is its pleiotropic functions as it is expressed on numerous other cells [18,20,22,23]. Thus, it is often difficult to ascertain that the results obtained from only using a *c-kit*-dependent mouse model are indeed due to MC deficiency rather than the influence of c-kit on other cell populations [13,21]. To account for this limitation, it has been stipulated to utilize a Cre/loxP mouse strain that is independent of a c-kit mutation while being MC-deficient through Cre-recombinase expression under the control of MC-specific promotors [13,21]. Therefore, employing additional genetically modified animals permit a more specific approach in targeting MC.

In general, when investigating complex biological responses, it is important to note that the MC function may overlap with other cells [13]. Vice versa, the more critical MC contributions are to insulin infusion and glycemic control, and it is likely that abnormalities are found in each of the different available MC-deficient strains utilized. As such, it is supported that the strongest conclusion about the importance of MCs is likely derived from investigations, which apply multiple model systems [13]. Thus, the study objective was to establish the sentinel role of MC contributions to IPP-induced tissue inflammation utilizing two different MC-deficient animal models: c-kit-dependent and c-kit-independent MC-deficient mice.

## 2. Materials and Methods

### 2.1. Animals and Air Pouch Generation

All studies were conducted with approval from the institutional animal care and use committee (IACUC) at Wayne State University. *C57BL/6J* and *c-KitW-sh/^W-Sh^* mast cell (MC)-deficient mice were purchased from Charles River and bred and maintained in-house. *C57BL/6-Cpa3-Cre*; *Mcl-l^fl/+^* breeding pairs were generously donated from Dr. Maurer at Charité Berlin Dermatology Centrum. Subsequently, MC-deficient *Cpa3-Cre*; *Mcl-1^fl/fl^* (also referred to as “Hello Kitty”) mice and the corresponding control (*Cpa3-Cre*; *Mcl-1^+/+^*) were obtained from these breeding pairs. Mice were maintained under temperature- and light-controlled conditions (20–24 °C, 12 h light-dark cycle) receiving food and water ad libitum. *Cpa3-Cre*; *Mcl-1^fl/fl^* mice, including control mice, were housed under pathogen-free conditions. All mice evaluated were between the ages of 6 and 8 weeks.

The air pouch model and its analysis have been described in previous publications [6,7,8,10]. Briefly, 3 mL of filtered air was injected subcutaneously into the shaved backs of the mice to create a sustained compartment one day prior to inserting the infusion cannulas. Infusion set cannulas (Animas Inset 30 Infusion System, ADW Diabetes, Pompano Beach, FL, USA) were implanted into the air pouch while mice were anesthetized and prior to beginning any infusions.

### 2.2. Assessment of IPP-Induced Inflammation in MC-Deficient Mouse Strains

Inflammation was assessed following the infusion of a sterile diluent (Eli Lilly & Co., Indianapolis, IN, USA) or Humalog^®^ U100 at a continuous rate of 50 µL/hour for 3 days. The sterile diluent (also referred as diluent) contains the phenolic preservatives phenol and m-cresol in a combined concentration of 2.25 mg/mL [7]. Humalog^®^ U100, with a concentration of 3.15 mg/mL m-cresol and trace amounts of phenol [7], was diluted at a concentration of 1 U/100 uL in sterile diluent. Control animals were infused with 0.9% saline solution. Diabetes was induced following the protocol developed by Wu et al. [24]. Streptozotocin (STZ) was prepared and administered as previously described [7]. Mice with a blood glucose level above 250 mg/dL for two sequential blood glucose tests were designated as diabetic. Once euglycemia was achieved, insulin infusion was replaced with diluent infusion to avoid hypoglycemic events. Furthermore, periodically switching between the infusion of diluted insulin versus diluent alone ensured that the same volume of fluid was infused between treatment groups while approximating euglycemia. A total of four to seven mice per treatment were used for each study.

### 2.3. Flow Cytometry Analysis

Flow analysis was conducted as previously described [7]. MC populations in the mouse air pouch were identified by gating c-Kit+ (2B8, Invitrogen, Waltham, MA, USA) and FceR1α+ (MAR-1, Invitrogen, Waltham, MA, USA) [25]. FACS analyses were performed on a BD LSR II utilizing the services of the microscopy, imaging, and cytometry core laboratory (MICR), at Wayne State University, Detroit, MI, USA, and data were analyzed with FlowJo software (v10, FlowJo™, LLC, Becton, Dickinson & Company, OR, USA).

### 2.4. Total Protein Analysis of Air Pouch Lavage Fluid

Lavage fluid collected from the air pouch was concentrated to 1 mL using MilliporeSigma™ Amicon™ Ultra-15 Centrifugal Filter Units (Fisher Scientific, Waltham, MA, USA). Protein quantification was performed on air pouch lavage fluid using the Pierce™ Coomassie (Bradford) Protein Assay Kit (Thermo Fisher Scientific, Waltham, MA, USA).

### 2.5. Immunohistochemistry

Qualitative immunostaining was performed on 5 µm 10% buffered-formalin (VWR, Radnor, PA)-fixed paraffin-embedded air pouch sections [7]. Sections were stained using standard Hematoxylin and Eosin stain (H&E). Mast cell presence was evaluated using toluidine blue (Sigma, St. Louis, MO, USA) [26]. The presence of macrophages was confirmed with antibodies against F4/80 (Fisher Scientific, Waltham, MA, USA). Mouse IgG was used as a negative control and analyzed by fluorescence microscopy (Nikon Instruments Inc., Melville, NY, USA).

### 2.6. Statistical Analysis

For multiple group comparisons, a one-way ANOVA test with a post-hoc Tukey test was utilized to assess differences at a 95% confidence interval. All statistical tests were performed, and the data were graphed using GraphPad Prism 8 software. The values were considered statistically significant for *p* < 0.05.

## 3. Results

### 3.1. Cpa3-Cre; Mcl-1^fl/fl^ Mice Exhibit a Marked Reduction in MCs in the Air Pouch Lavage

**Figure 1:**
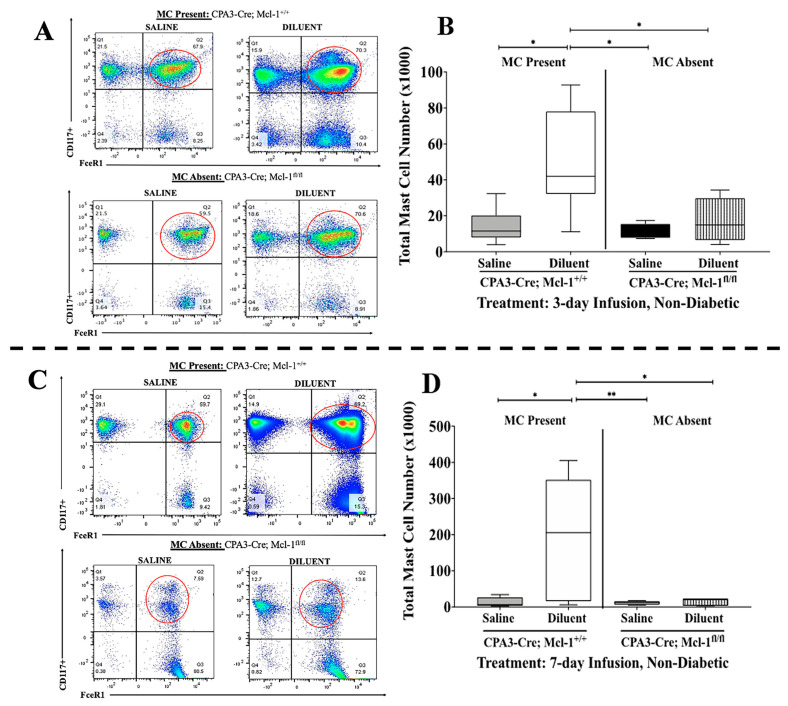
Assessment of MC influx into the air pouch following the infusion of saline or diluent. (**A**,**C**) Representative flow cytometry plots showing comparable expression of MCs in the air pouch lavage collected from *Cpa3-Cre*; *Mcl-1^+/+^* and *Cpa3-Cre*; *Mcl-1^fl/fl^* mice (*FcεRI+*; *c-Kit+*, red circle indicates MC population) following 3- and 7-day infusion, respectively. (**B**) MC quantification in air pouch lavage following saline and diluent infusion in *Cpa3-Cre*; *Mcl-1^+/+^* mice and in *Cpa3-Cre*; *Mcl-1^fl/fl^* mice for 3 days. (**D**) MC quantification of air pouch lavage following saline and diluent infusion in *Cpa3-Cre*; *Mcl-1^+/+^* mice and in *Cpa3-Cre*; *Mcl-1^fl/fl^* mice for 7 days. * *p* < 0.05, ** *p* < 0.001 using one-way ANOVA with Tukey HDS multiple comparisons test.

The *Cpa3-Cre*; *Mcl-1^fl/fl^* mice were first introduced more than a decade ago by Galli et al. [25]. Nonetheless, no data are available related to the impact of insulin preservatives on the subcutaneous tissue using this mouse strain. Thus, we used flow cytometry to first assess the mast cell (MC) presence in the air pouch fluid following saline and diluent, a phenolic compound, infusion over 3 and 7 days. *Cpa3-Cre*; *Mcl-1^fl/fl^* mice exhibited significant reductions in MC numbers in the diluent-infused air pouch for both 3 and 7 days (Figure 1). In contrast, MCs were present in similar numbers for control *Cpa3-Cre*; *Mcl-1^+/+^* mice and the Cre/lox MC strain for the saline control fluid for both time points.

### 3.2. MC-Deficient Mice Exhibit Evidently Reduced Leukocyte Recruitment in the Air Pouch

**Figure 2:**
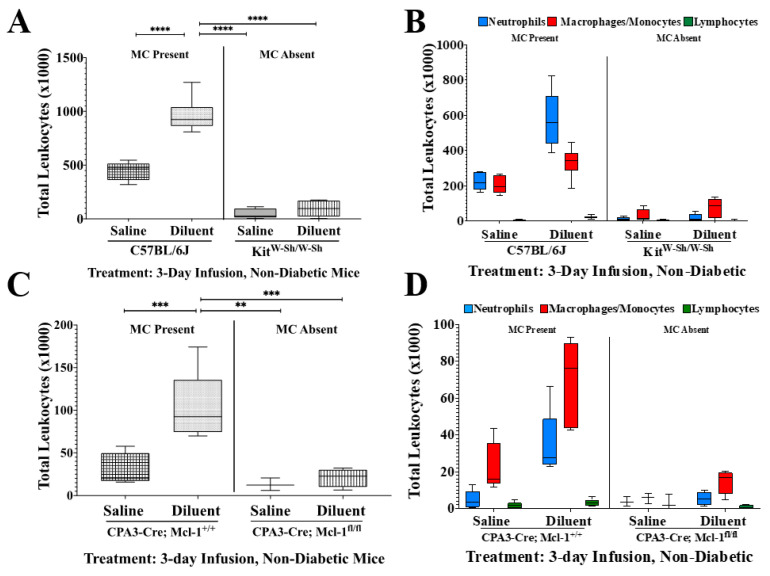
Impact of mast cell (MC)-deficiency on total leukocyte influx and leukocyte subpopulations following infusion for 3 days into the air pouch of non-diabetic animals. (**A**) Quantitative analysis of the total leukocytes presence in the air pouch of *C57BL/6J* control and *c-Kit^W-sh/W-Sh^* MC-deficient mice infused with saline and diluent for 3 days. (**B**) Quantification of the leukocyte subpopulations PMNs, MQs/Mos, and lymphocytes following saline and diluent infusion in *C57BL/6J* and *c-Kit^W-sh/W-Sh^* MC-deficient mice. (**C**) Quantitative analysis of the total leukocytes present in the air pouch of *Cpa3-Cre*; *Mcl-1^+/+^* control and *Cpa3-Cre*; *Mcl-1^fl/fl^* MC-deficient mice infused with saline and diluent for 3 days. (**D**) Quantification of the leukocyte subpopulations PMNs, MQs/Mos, and lymphocytes following infusion of saline and diluent into *Cpa3-Cre*; *Mcl-1^+/+^* control and *Cpa3-Cre*; *Mcl-1^fl/fl^* MC-deficient mice. ** *p* < 0.01, *** *p* < 0.001, **** *p* < 0.0001 using one-way ANOVA with Tukey HDS multiple comparisons test. Statistical analyses of individual leukocyte subpopulations can be found in Table 1 and Table 2.

**Figure 3:**
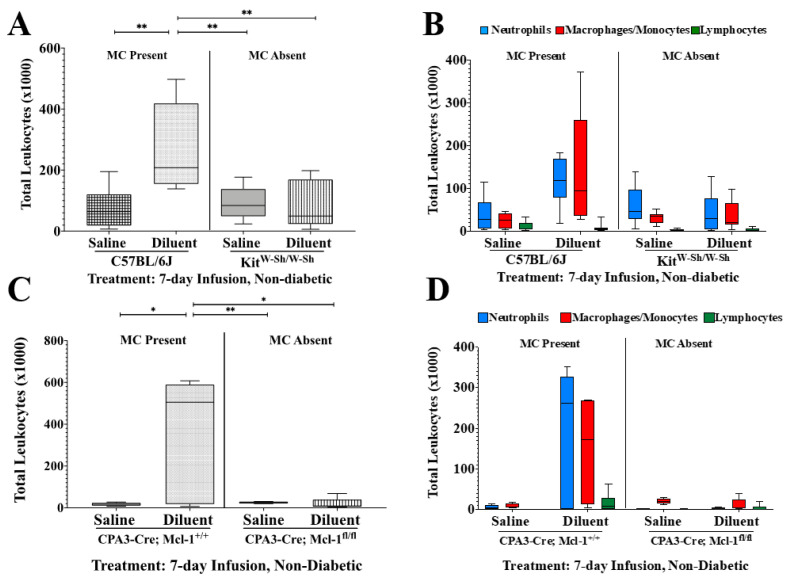
Impact of mast cell (MC) deficiency on total leukocyte influx and leukocyte subpopulations following infusion for 7 days into the air pouch of non-diabetic animals. (**A**) Quantitative analysis of the total leukocytes present in the air pouch of *C57BL/6J* control and *c-Kit^W-sh/W-Sh^* MC-deficient mice infused with saline and diluent for 7 days. (**B**) Quantification of the leukocyte subpopulations PMNs, MQs/Mos, and lymphocytes following the infusion of saline and diluent into *C57BL/6J* and *c-Kit^W-sh/W-Sh^* MC-deficient mice. (**C**) Quantitative analysis of the total leukocytes present in the air pouch of *Cpa3-Cre*; *Mcl-1^+/+^* control and *Cpa3-Cre*; *Mcl-1^fl/fl^* MC-deficient mice infused with saline and diluent for 7 days. (**D**) Quantification of the leukocyte subpopulations PMNs, MQs/Mos, and lymphocytes following the infusion of saline and diluent into *Cpa3-Cre*; *Mcl-1^+/+^* control and *Cpa3-Cre*; *Mcl-1^fl/fl^* MC-deficient mice. * *p* < 0.05, ** *p* < 0.01 using one-way ANOVA with Tukey HDS multiple comparisons test. Statistical analyses of leukocyte subpopulations can be found in Table 1 and Table 2.

To investigate the role of MC deficiency in leukocyte recruitment following the infusion of diluent, we performed flow cytometry assessing total leukocyte and leukocyte subpopulation recruitment into the mouse air pouch over 3 and 7 days (Figure 2 and Figure 3, including Table 1 and Table 2). We observed statistically significant reductions in total leukocyte numbers for the Kit-dependent, *Kit^W-Sh^*, and the Kit-independent, *Cpa3-Cre*; *Mcl-1^fl/fl^*, MC-deficient mice, as compared to the control mice, *Cpa3-Cre*; *Mcl-1^+/+^* and *C57BL/6J*, for both time points during diluent infusion. Also, there were significantly fewer leukocytes following saline infusion in the *C57BL/6J* and *Cpa3-Cre*; *Mcl-1^+/+^* mice as compared to with diluent infusion in those same mice, as depicted in Figure 2 and Figure 3, for 3-day and 7-day infusion studies, respectively.

Cellular subtype analyses following 7-day infusion in non-diabetic mice (Figure 3) showed significantly fewer neutrophils (PMNs) following diluent infusion in the Kit^W-Sh^ group as compared to numbers following diluent infusion in the *C57BL/6J* mice (*p* < 0.05). The PMNs quantified from *Cpa3-Cre*; *Mcl-1^fl/fl^* mice infused with saline and diluent were significantly less abundant than PMNs following the infusion of diluent in the CPA3-Cre; Mcl-1^+/+^ mice (*p* < 0.05 and *p* < 0.01, respectively). Also, PMN presence was significantly reduced in both control mouse air pouch tissue infused with saline as compared to that with diluent (*p* < 0.05). Upon 7-day infusion, macrophages/monocytes (MQs/Mos) were significantly less abundant following saline and diluent infusion in the Kit^W-Sh^ mice as compared to numbers with diluent infusion in *C57BL/6J* mice (*p* < 0.05). In the *Cpa3-Cre*; *Mcl-1^fl/fl^* mice, MQ/Mo presence was significantly lower following only diluent infusion as compared to that with diluent infusion in the *CPA3-Cre*; *Mcl-1^+/+^* control mice (*p* < 0.05). There was no significant difference in the lymphocyte population among any of the treatment groups following the 7-day infusion (Table 1 and Table 2).

### 3.3. Total Protein Analysis of Lavage Fluid following Saline and Diluent Infusion

**Figure 4:**
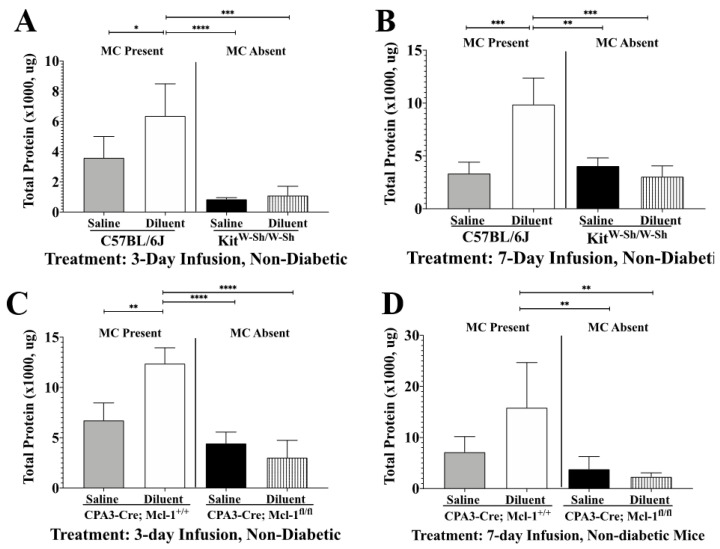
Impact of mast cell (MC) deficiency on total protein present in the air pouch fluid following infusion for 3 and 7 days into the air pouch of non-diabetic animals. Quantification of total protein present in the air pouch fluid following the infusion of saline and diluent for 3 days (**A**,**C**) and 7 days (**B**,**D**) into the air pouch of control mice (*C57BL/6J* and *Cpa3-Cre*; *Mcl-1^+/+^)* and MC-deficient mice (*c-Kit^W-sh/W-Sh^* and *Cpa3-Cre*; *Mcl-1^fl/fl^*). * *p* < 0.05, ** *p* < 0.01, *** *p* < 0.001, **** *p* < 0.0001, using one-way ANOVA with Tukey HDS multiple comparisons test.

Previous studies have indicated that IPP-induced inflammation leads to edema in the air pouch tissue of mice following infusion for up to 7 days [10]. To evaluate the impact of MCs on edema following IPP infusion, total protein in the lavage fluid was quantified following the infusion of either saline or diluent into the air pouch of non-diabetic mice (Figure 4). The infusion of diluent over a 3-day period into the air pouch of control *C57BL/6J* mice revealed significantly more total protein as compared to that with the infusion of saline or diluent into Kit^W-Sh^ mice (Figure 4A, *p* < 0.0001 and *p* < 0.001, respectively). Similarly, when the air pouch of *Cpa3-Cre*; *Mcl-1^fl/fl^* mice was infused with saline or diluent, there was significantly less total protein present when compared to diluent infusion of the control mice *CPA3-Cre*; *Mcl-1^+/+^* (Figure 4C, *p* < 0.0001). Furthermore, there was significantly less total protein following the 3-day saline infusion when compared to that with 3-day diluent infusion from both control mice, *C57BL/6J* and *CPA3-Cre*; *Mcl-1^+/+^* (Figure 4A, *p* < 0.05 and *p* < 0.01, respectively). A similar pattern was seen when evaluating the lavage fluid following the 7-day infusion (Figure 4B,D). Saline and diluent infusion into the air pouch of Kit^W-Sh^ mice resulted in significantly less total protein present when compared to diluent infusion into the *C57BL/6J* air pouch (Figure 4B, *p* < 0.01 and *p* < 0.001, respectively). Similarly, saline and diluent infusion into the *Cpa3-Cre*; *Mcl-1^fl/fl^* mice led to significantly less total protein when compared to diluent infusion into the control mice, *CPA3-Cre*; *Mcl-1^+/+^* (Figure 4D, *p* < 0.01).

### 3.4. Kit^W-Sh^ and Cpa3-Cre; Mcl-1^fl/fl^ MC-Deficient Mice Exhibit Reduced Tissue Reactions following Phenolic Preservative Infusion

**Figure 5:**
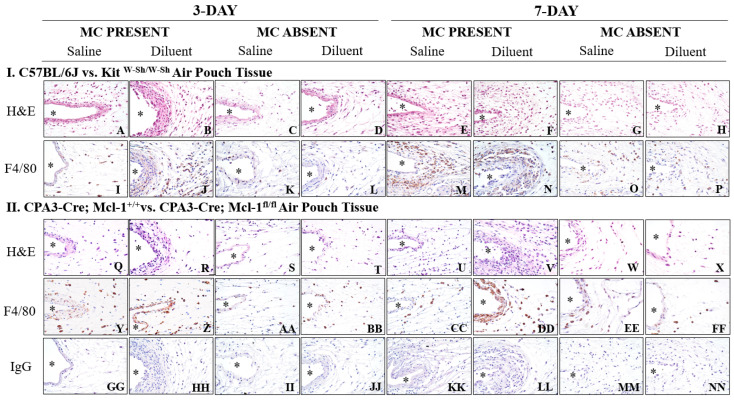
Histopathologic evaluation of mouse air pouch tissue following the infusion of saline and diluent into the control and MC-deficient non-diabetic mice. To evaluate the tissue reactions following the infusion of either saline or diluent into the air pouch of various mouse models, the mouse air pouch tissue was collected at the end of 3- and 7-day infusions (post-lavage). Standard hematoxylin (H&E) staining was performed on the c-kit-dependent MC-deficient mice *Kit ^W-Sh/W-Sh^* and controls *C57BL/6J (***A**–**H**), as well as the c-kit-independent MC-deficient mice, *CPA3-Cre*; *Mcl-1^fl/fl^*, and controls, *CPA3-Cre*; *Mcl-1^+/+^* (**Q**–**X**). To evaluate the presence and distribution of macrophages, a macrophage-specific F4/80 antibody was utilized (**I**–**P**, **Y**–**FF**). Controls of normal IgG are also represented (**GG**–**NN**). The location of the air pouch is designated by (*). All images are 40× magnification.

To determine whether MC deficiency resulted in a reduced tissue response to the continuous air pouch infusion of saline or diluent, we evaluated the air pouch tissue using standard histopathology techniques (Figure 5). Histopathological evaluation revealed that saline infusion for 3 days into the air pouch of the control mice (*C57BL/6J* and *CPA3-Cre*; *Mcl-1^+/+^)* resulted in the light scattering of inflammatory cells near the air pouch interface (Figure 5A,Q). However, diluent infusion into the control mice resulted in a greater number of inflammatory cells directly near the air pouch interface and spreading out to the adjoining tissue (Figure 5B,R). Saline and diluent infusion into the air pouch of both MC-deficient mouse strains (*Kit^W-Sh^* and *Cpa3-Cre*; *Mcl-1^fl/fl^*) revealed comparable tissue responses to the saline infusion into the C57BL/6J control mouse strain (Figure 5C–D,S–T). This was evident based on the minimal number of inflammatory cells near the air pouch and surrounding tissue. Most of the accumulating inflammatory cells were PMNs and macrophages (MQs), as confirmed by H&E and F4/80 immunohistochemical staining. PMNs are F4/80 negative but were confirmed morphologically using H&E analysis. The MQ presence was limited following saline and diluent infusion into the air pouch for the *Kit^W-Sh^* and *Cpa3-Cre*; *Mcl-1^fl/fl^* mice (Figure 5K,L,AA,BB). There was a greater presence of MQs near the air pouch and surrounding tissue following diluent infusion into the air pouch of mice that were MC-sufficient (Figure 5J,Z).

Overall, the tissue response following the 7-day infusion of saline and diluent into the MC-deficient mice (Figure 5G,H,W,X) was similar to the 3-day tissue response of these mice. The presence of MQ in this tissue was also minimal with a few MQs scattered through the air pouch tissue (Figure 5O,P,EE,FF). However, there was a more prominent MQ presence in the *C57BL/6J* mice near the air pouch and surrounding tissue following the 7-day infusion of diluent (Figure 5N,DD). Saline infusion into the *C57BL/6J* mice for 7 days resulted in slightly more inflammatory cells, including MQs, near the air pouch when compared to 3 days (Figure 5E,M,U,CC). Normal IgG did not result in any specific or non-specific staining for all treatments (Figure 5GG–NN).

### 3.5. Insulin Infusion into Air Pouch Does Not Augment Leukocyte Influx

**Figure 6:**
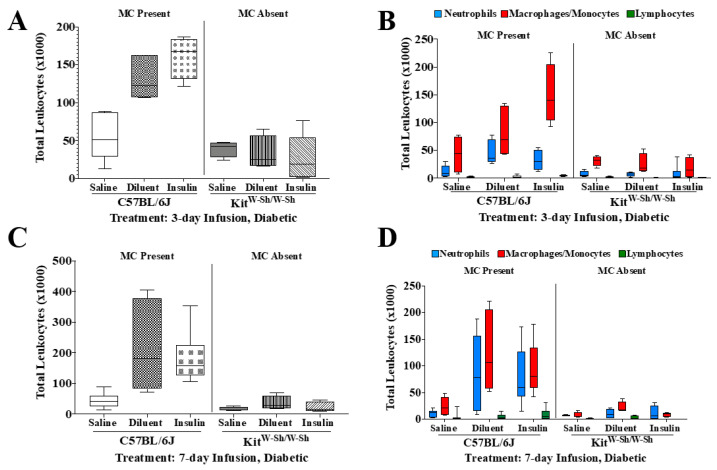
Impact of mast cell (MC) deficiency on total leukocyte influx and leukocyte subpopulations following infusion for 3 and 7 days into the air pouch of diabetic animals. (**A**) Quantitative analysis of the total leukocytes present in the air pouch of *C57BL/6J* control and *c-Kit^W-sh/W-Sh^* MC-deficient mice infused with saline, diluent, and insulin for 3 days. (**B**) Quantification of the leukocyte subpopulations PMNs, MQs/Mos, and lymphocytes following the infusion of saline, diluent, and insulin into *C57BL/6J* control and *c-Kit^W-sh/W-Sh^* MC-deficient mice. (**C**) Quantitative analysis of the total leukocyte presence in the air pouch of *C57BL/6J* control and *c-Kit^W-sh/W-Sh^* MC-deficient mice infused with saline, diluent, and insulin for 7 days. (**D**) Quantification of the leukocyte subpopulations PMNs, MQs/Mos, and lymphocytes following the infusion of saline and diluent into *Cpa3-Cre*; *Mcl-1^+/+^* control and *Cpa3-Cre*; *Mcl-1^fl/fl^* MC-deficient mice. Statistical analyses can be found in Table 3 and Table 4.

As it is well known that wound-healing defects are aberrant in diabetes [27,28], we investigated leukocyte influx into the air pouch of *C57BL/6J*- and *Kit^W-Sh^*-STZ-induced diabetic mice infused with either saline, diluent, or insulin (Figure 6, Table 3 and Table 4). The STZ-induced *C57BL/6J* and *Kit^W-Sh^* mice showed similar leukocyte influx patterns when compared to those in the non-diabetic group for both time points, 3 and 7 days. As was the case for non-diabetic mice, saline infusion led to a significantly lower total leukocyte presence when compared to that with diluent and insulin infusion into the air pouch of *C57BL/6J* diabetic mice (Figure 6A,C). Similarly, saline air pouch infusion into *Kit^W-Sh^* diabetic mice led to significantly less overall leukocyte recruitment as compared to that with diluent or insulin infusion into the air pouch of *C57BL/6J* diabetic mice. Diluent and insulin infusion into diabetic *C57BL/6J* mice led to significantly more leukocyte recruitment when compared to that with diluent or insulin infusion into *Kit^W-Sh^* diabetic mice (Figure 6A,C).

Leukocyte subpopulation analysis of PMNs, MQs/Mos, and lymphocytes was performed on 3- and 7-day infusions for both *C57BL/6J* and *Kit^W-Sh^* STZ-induced diabetic mice (Figure 6B,D and Table 3 and Table 4). There were significantly fewer PMNs recruited to the air pouch following saline infusion into the *C57BL/6J* diabetic mice as compared to with diluent infusion into the same mice for both time points (*p* < 0.05). Saline infusion into the air pouch of *Kit^W-Sh^* diabetic mice led to a significantly lower PMN presence when compared to diluent infusion into *C57BL/6J* mice over 3 days (*p* < 0.01), but no statistical significance was observed for the 7-day infusion. However, a different trend was observed for the MQ/Mo presence between the two time-points. Specifically, there were significantly fewer total MQs/Mos following the infusion of saline into diabetic *C57Bl/6J* mice when compared to numbers with insulin infusion in the same mice for both time points (*p* < 0.01 and *p* < 0.05, respectively). Furthermore, saline infusion into *Kit^W-Sh^* diabetic mice led to significantly fewer total MQs/Mos as compared to numbers with insulin infusion into *C57BL/6J* mice for 3 and 7 days (*p* < 0.001 and *p* < 0.05, respectively). Diluent and insulin infusion into *C57BL/6J* diabetic mice led to significantly greater MQ/Mo recruitment to the air pouch when compared to that with the insulin infusion of MC-deficient mice for 3 days (*p* < 0.05 and *p* < 0.0001, respectively) and 7 days (*p* < 0.01 and *p* < 0.05, respectively). Also, insulin infusion into *C57BL/6J* diabetic mice resulted in a significantly greater MQ/Mo presence as compared to that with diluent infusion into MC-deficient mice for 3 days (*p* < 0.001). Lymphocyte analysis was also performed among the treatment groups, and there were significantly more lymphocytes recruited to the air pouch following insulin infusion into *C57BL/6J* mice as compared to with diluent or insulin infusion into MC-deficient mice for the 3-day infusion (*p* < 0.05). There were no significant differences in lymphocyte recruitment among any of the treatment groups for 7 days (Table 4).

### 3.6. Insulin Infusion Does Not Intensify Inflammation

**Figure 7:**
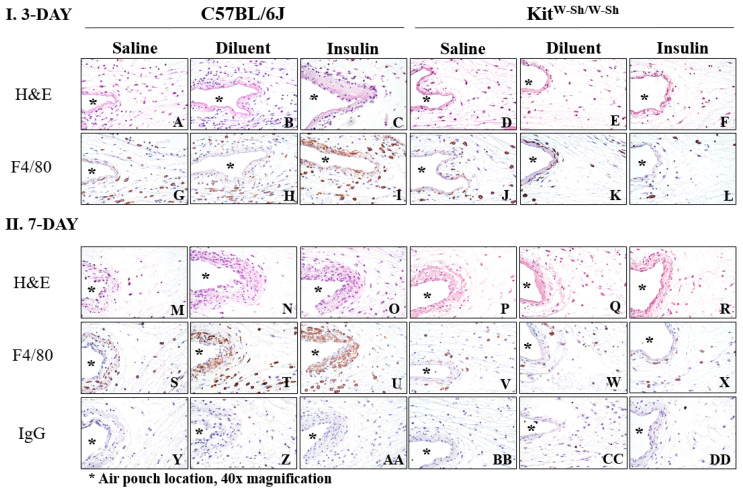
Histopathologic evaluation of mouse air pouch tissue following the infusion of saline, diluent, and insulin into control and MC-deficient diabetic mice. To evaluate the tissue reactions following the infusion of either saline, diluent, or insulin into the air pouch of diabetic MC-deficient mice and controls, the mouse air pouch tissue was collected at the end of 3- and 7-day infusions (post-lavage). Standard hematoxylin (H&E) staining was performed on the c-kit-dependent MC-deficient mice, *Kit ^W-Sh/W-Sh^*, and controls, *C57BL/6J*, following the 3-day infusion (**A**–**F**) and 7-day infusion (**M**–**R**). To evaluate the presence and distribution of macrophages, a macrophage specific F4/80 antibody was utilized (**G**–**L**, **S**–**X**). Controls of normal IgG are also represented (**Y**–**DD**). The location of the air pouch is designated by (*). All are 40× magnification.

Using non-diabetic mice, we demonstrated that both MC-deficient strains, *Kit^W-Sh^* and *Cpa3-Cre*; *Mcl-1^fl/fl^*, experienced significantly fewer tissue reactions than control mice (*C57BL/6J* and *CPA3-Cre*; *Mcl-1^+/+^)* during diluent infusion. Thus, we investigated if insulin infusion would augment the tissue reaction using STZ-induced diabetic *C57BL/6J* and *Kit^W-Sh^* mice. These diabetic mice were infused with saline, diluent, or insulin for 3 days and 7 days continuously, and the same histological analyses were performed on the air pouch tissue post-infusion as was accomplished on the non-diabetic air pouch tissue (Figure 7). Diluent and insulin infusion for 3 days into the air pouch of the diabetic control mice (*C57BL/6J*) led to greater numbers of inflammatory cells, specifically neutrophils (PMNs) and macrophages (MQ), adjacent to the air pouch and surrounding tissue as compared to those in the diabetic MC-deficient mouse air pouch tissue (*Kit^W-Sh^*) (Figure 7B–F). Saline infusion into the control mice was comparable to the MC-deficient mice with limited number of inflammatory cells near the air pouch (Figure 7A). The MQ presence was also greater in the control mice infused with diluent and insulin, with MQs present directly at the air pouch interface and in the surrounding tissue (Figure 7H,I). However, MQs were limited in the MC-deficient air pouch tissue infused with saline, diluent, and insulin, as well as in the control tissue infused with saline (Figure 7G,J–L).

Overall, there was a similar pattern of the inflammatory cell presence following the 7-day infusion into the diabetic mice. However, there were notably more MQs present in the tissue of the control mice. More specifically, the infusion of diluent and insulin into diabetic control mice led to a more pronounced band of inflammatory cells near the air pouch, as well as a greater number of MQs directly near the air pouch (Figure 7N,O,T,U). Furthermore, diluent and insulin infusion into the control mice led to the substantial presence of proteinaceous material (e.g., red staining material in H&E-stained slides) (Figure 7N,O). The infusion of saline, diluent, and insulin into the MC-deficient mice for 7 days led to a similar tissue response to that with the 3-day infusion with a limited number of inflammatory cells and MQs at and near the air pouch (Figure 7Q,R,W,X). Normal IgG did not show any specific or non-specific staining for all treatment groups (Figure 7Y–DD). Overall, we did not observe any differences in tissue reactions when comparing insulin infusion versus phenolic preservative (e.g., diluent) infusion.

## 4. Discussion

This study elucidated the role of MCs in IPP-induced inflammation during subcutaneous insulin infusion using *c-kit*-dependent and c-kit-independent MC-deficient mice, including a murine air pouch model. These studies used a genetic approach designed to analyze MC functions in IPP-induced inflammation. MC-deficient mice with a mutation in *KIT*, such as *C57BL/6-Kit^W-sh/W-sh^*, have been extensively used to study the role of MCs in disease pathogenesis and wound healing [13,29,30,31]. The Cre-loxP recombination system provides MC-deficiency, yet it lacks the abnormalities related to the c-kit expression and structure [25,32,33]. Thus, this additional mouse model was chosen to investigate leukocyte recruitment following continuous infusions for 3 and 7 days in a mouse air pouch through flow cytometry analysis of the cell lavage contents, including histopathologic analysis. Notably, two independent MC-deficient mouse strains, the *Cpa3-Cre*; *Mcl-1^fl/fl^* and the *C57BL/6j-Kit^W-Sh/W-sh^*, demonstrated the same outcome. Thus, we conclude that obtaining the same results using a pharmacological approach [11] and a genetic approach while utilizing two distinctive different MC-deficient mouse strains establishes an MC role in IPP-induced inflammation.

The histopathological evaluations of the air pouch post-lavage indicated that MC absence led to substantially less leukocyte recruitment following IPP infusion. More specifically, the presence of MQs/Mos and PMNs near the air pouch interface was noticeably diminished when diluent was infused into the MC-deficient mice (Figure 5). Furthermore, in the control mice, which are MC sufficient, edema was evident following diluent infusion as evidenced by the pink-stained band directly near the air pouch. This edema was substantially reduced in the air pouch tissue collected from the MC-deficient mice. This is further supported by the protein content quantified from the air pouch fluid (Figure 4). The protein content in the lavage fluid collected from the MC-deficient mice was significantly less than that in the lavage fluid collected from the control mice (Figure 4). This lends further credence to our hypothesis that MC presence during IPP-induced inflammation incites acute inflammation that is characterized by edema and leukocyte infiltration. These data correlate with other MC-deficient studies, which showed reduced leukocyte recruitment in response to a stimulus [25]. This was expected as MCs contribute to neutrophil recruitment while concomitantly synthesizing chemokines to operate in synergy with MQs, permitting leukocyte migration deep into the inflamed tissue site [34]. MCs are known to release CCL-2/MCP-1, a strong chemoattractant for monocytic cells [35]. In addition, MC and macrophage interaction occurs through the MC surface receptors FcεRI and MQ caspase-1 expression, which is up-regulated following contact with MCs [36]. As our lavage data demonstrated that MQs are one of the dominant cells at the site of IPP infusion sets, future studies are directed to gain a better understanding of the role of mast cell–macrophage interactions and how they may contribute to the tissue reaction at the site of subcutaneous insulin administration. Future studies should also investigate specific pro-inflammatory mediators distributed following IPP-induced MC activation. This will provide important information regarding the specific chemokines and cytokines released, including their role in pro-inflammatory pathways.

Diabetic patients experience an increase in MC degranulation and suboptimal wound healing [27,28,37]. Thus, we investigated the role of the MC presence in diabetic animals in *Kit^W-Sh^* STZ-induced diabetic mice, including a control strain, during infusion studies. These studies demonstrated that a lack of MCs in diabetic mice is associated with reduced leukocyte recruitment following the infusion of the preservative diluent and the insulin Humalog^®^ for days 3 and 7. The histopathological evaluation of the diabetic air pouch tissue following the infusion of diluent or Humalog^®^ indicated substantially fewer inflammatory cells near the air pouch interface as compared to numbers in the air pouch tissue from the diabetic control mice. Furthermore, the use of a macrophage-specific antibody, F4/80, indicated that the macrophage presence was considerably decreased in the air pouch tissue of diabetic mice deficient in MCs and when infused with diluent or Humalog^®^. This further supports the hypothesis that IPP activates MCs, which leads to chronic inflammation that may be characterized by macrophage presence. Of note is the significantly increased total leukocyte count mainly driven by an increased neutrophil influx at 3 days in the air pouch of non-diabetic control mice (*C57BL/*6J) (Figure 2A,B). This was not repeated at day 7 or in the *HSD:CD-1 strains* [6,7,8,10,11]. Although, reduced PMN chemotaxis [38], including the reduced leukocyte recruitment in diabetes patients [39] compared to that in non-diabetic controls, has been demonstrated, the reduced immune response in IPP-induced leukocyte influx in STZ-induced diabetic *C57BL/6*J mice over a 3-day period warrants further investigation.

## 5. Conclusions

Concordant results using two independent MC-deficient mouse models establishes an MC role in IPP-induced infusion. Furthermore, these studies indicate the therapeutic potential of targeting MCs to attenuate insulin preservative-induced inflammation ensuring healthy tissue at the site of prolonged insulin administration.

## Figures and Tables

**Table 1:** Statistical analysis of flow cytometry data following 3- and 7-day infusions in non-diabetic kit-dependent MC-deficient mice and controls (*Kit^W-Sh/W-Sh^ and C57BL6/J*).

Tukey’s Multiple Comparisons Test		Total Cells		PMNs		MQ/Mo		LYMPH	
		Summary		Summary		Summary		Summary	
Group 1	Group 2	3-Day	7-Day	3-Day	7-Day	3-Day	7-Day	3-Day	7-Day
Saline C57BL/6J	Saline Kit ^W-Sh/W-Sh^	****	ns	*	ns	**	ns	ns	ns
Saline C57BL6	Diluent C57BL6	****	**	****	*	*	ns	***	ns
Saline C57BL6	Diluent Kit ^W-Sh/W-Sh^	***	ns	*	ns	*	ns	ns	ns
Saline Kit ^W-Sh/W-Sh^	Diluent C57BL6	****	**	****	ns	****	*	****	ns
Saline Kit ^W-Sh/W-Sh^	Diluent Kit ^W-Sh/W-Sh^	ns	ns	ns	ns	ns	ns	ns	ns
Diluent C57BL6	Diluent Kit ^W-Sh/W-Sh^	****	**	****	*	****	*	****	ns

Statistical significance for flow cytometry analysis of total cells, neutrophils (PMNs), macrophages/monocytes (MQs/Mos), and lymphocytes (lymph) following 3-day and 7-day infusion of saline or diluent into the air pouch of non-diabetic MC-deficient mice, *Kit^W-Sh/W-Sh^* and control mice *C57BL/6J*. Analyses performed with one-way ANOVA and a Tukey post-hoc multiple comparison test. * *p* < 0.05, ** *p* < 0.01, *** *p* < 0.001, **** *p* < 0.0001, ns = not significant.

**Table 2:** Statistical analysis of 3- and 7-day infusion in non-diabetic kit-independent MC-deficient mice and controls (*Cpa3-Cre*; *Mcl-1^fl/fl^* and *Cpa3-Cre*; *Mcl-1^+/+^*).

Tukey’s Multiple Comparisons Test		Total Cells		PMNs		MQ/Mo		LYMPH		MC	
		Summary		Summary		Summary		Summary		Summary	
Group 1	Group 2	3-Day	7-Day	3-Day	7-Day	3-Day	7-Day	3-Day	7-Day	3-Day	7-Day
Saline CPA3-Cre; Mcl-1^+/+^	Saline CPA3-Cre; Mcl-1^fl/fl^	ns	ns	ns	ns	ns	ns	ns	ns	ns	ns
Saline CPA3-Cre; Mcl-1^+/+^	Diluent CPA3-Cre; Mcl-1^+/+^	***	*	**	*	***	*	ns	ns	*	ns
Saline CPA3-Cre; Mcl-1^+/+^	Diluent Cpa3-Cre; Mcl-1^fl/fl^	ns	ns	ns	ns	ns	ns	ns	ns	ns	ns
SalineCpa3-Cre; Mcl-1^fl/fl^	Diluent CPA3-Cre; Mcl-1^+/+^	***	*	**	*	***	ns	ns	ns	*	ns
Saline Cpa3-Cre; Mcl-1^fl/fl^	Diluent Cpa3-Cre; Mcl-1^fl/fl^	ns	ns	ns	ns	ns	ns	ns	ns	ns	ns
Diluent CPA3-Cre; Mcl-1^+/+^	Diluent Cpa3-Cre; Mcl-1^fl/fl^	***	**	**	**	***	*	ns	ns	*	*

Statistical significance for FACS analysis of total cells, neutrophils (PMNs), macrophages/monocytes (MQs/Mos), lymphocytes (lymph), and MCs (MC) following the 3-day and 7-day infusion of saline or diluent into the air pouch of non-diabetic MC-deficient mice, *Cpa3-Cre*; *Mcl-1^fl/fl^*, and control mice, *Cpa3-Cre*; *Mcl-1^+/+^.* Analyses were performed with one-way ANOVA and a Tukey post-hoc multiple comparison test. * *p* < 0.05, ** *p* < 0.01, *** *p* < 0.001, ns = not significant.

**Table 3:** Statistical analysis of 3-day infusion in diabetic kit-dependent MC-deficient mice and controls (*C57BL6/J* vs*. Kit ^W-Sh/W-Sh^*).

Tukey’s Multiple Comparisons Test		Total Cells	PMNs	MQ/Mo	LYMPH
Group 1	Group 2	Summary	Summary	Summary	Summary
Saline C57BL/6J	Saline Kit ^W-Sh/W-Sh^	ns	ns	ns	ns
Saline C57BL/6J	Diluent C57BL/6J	*	*	ns	ns
Saline C57BL/6J	Diluent Kit ^W-Sh/W-Sh^	ns	ns	ns	ns
Saline C57BL/6J	Insulin C57BL/6J	***	ns	**	ns
Saline C57BL/6J	Insulin Kit ^W-Sh/W-Sh^	ns	ns	ns	ns
Saline Kit ^W-Sh/W-Sh^	Diluent C57BL/6J	*	**	ns	ns
Saline Kit ^W-Sh/W-Sh^	Diluent Kit ^W-Sh/W-Sh^	ns	ns	ns	ns
Saline Kit ^W-Sh/W-Sh^	Insulin C57BL/6J	***	ns	***	ns
Saline Kit ^W-Sh/W-Sh^	Insulin Kit ^W-Sh/W-Sh^	ns	ns	ns	ns
Diluent C57BL/6J	Diluent Kit ^W-Sh/W-Sh^	**	**	ns	ns
Diluent C57BL/6J	Insulin C57BL/6J	ns	ns	ns	ns
Diluent C57BL/6J	Insulin Kit ^W-Sh/W-Sh^	**	**	*	ns
Diluent Kit ^W-Sh/W-Sh^	Insulin C57BL/6J	****	ns	***	*
Diluent Kit ^W-Sh/W-Sh^	Insulin Kit ^W-Sh/W-Sh^	ns	ns	ns	ns
Insulin C57BL/6J	Insulin Kit ^W-Sh/W-Sh^	****	ns	****	*

Statistical significance for FACS analysis of total cells, neutrophils (PMNs), macrophages/monocytes (MQs/Mos), and lymphocytes (lymph) following the 3-day infusion of saline, diluent, and insulin into the air pouch of diabetic MC-deficient mice, *Kit ^W-Sh/W-Sh^*, and control mice, *C57BL/6J*. Analyses were performed with one-way ANOVA and a Tukey post-hoc multiple comparison test. * *p* < 0.05, ** *p* < 0.01, *** *p* < 0.001, **** *p* < 0.0001, ns = not significant.

**Table 4:** Statistical analysis of 7-day infusion in diabetic kit-dependent MC-deficient mice and controls (*C57BL6/J* vs. *Kit ^W-Sh/W-Sh^*).

Tukey’s Multiple Comparisons Test		Total Cells	PMNs	MQ/Mo	LYMPH
Group 1	Group 2	Summary	Summary	Summary	Summary
Saline C57BL/6J	Saline Kit ^W-Sh/W-Sh^	ns	ns	ns	ns
Saline C57BL/6J	Diluent C57BL/6J	**	*	**	ns
Saline C57BL/6J	Diluent Kit ^W-Sh/W-Sh^	ns	ns	ns	ns
Saline C57BL/6J	Insulin C57BL/6J	*	ns	*	ns
Saline C57BL/6J	Insulin Kit ^W-Sh/W-Sh^	ns	ns	ns	ns
Saline Kit ^W-Sh/W-Sh^	Diluent C57BL/6J	**	ns	**	ns
Saline Kit ^W-Sh/W-Sh^	Diluent Kit ^W-Sh/W-Sh^	ns	ns	ns	ns
Saline Kit ^W-Sh/W-Sh^	Insulin C57BL/6J	*	ns	*	ns
Saline Kit ^W-Sh/W-Sh^	Insulin Kit ^W-Sh/W-Sh^	ns	ns	ns	ns
Diluent C57BL/6J	Diluent Kit ^W-Sh/W-Sh^	*	ns	**	ns
Diluent C57BL/6J	Insulin C57BL/6J	ns	ns	ns	ns
Diluent C57BL/6J	Insulin Kit ^W-Sh/W-Sh^	*	*	**	ns
Diluent Kit ^W-Sh/W-Sh^	Insulin C57BL/6J	ns	ns	ns	ns
Diluent Kit ^W-Sh/W-Sh^	Insulin Kit ^W-Sh/W-Sh^	ns	ns	ns	ns
Insulin C57BL/6J	Insulin Kit ^W-Sh/W-Sh^	*	ns	*	ns

Statistical significance for FACS analysis of total cells, neutrophils (PMNs), macrophages/monocytes (MQs/Mos), and lymphocytes (lymph) following the 7-day infusion of saline, diluent, and insulin into the air pouch of diabetic MC-deficient mice, *Kit ^W-Sh/W-Sh^*, and control mice, *C57BL/6J*. Analyses were performed with one-way ANOVA and a Tukey post-hoc multiple comparison test. * *p* < 0.05, ** *p* < 0.01, ns = not significant.

## Data Availability

Data are contained within the article. The data presented in this study are available on request from the corresponding author.

## References

[1] Forlenza G.P., Lal R.A. (2022). Current Status and Emerging Options for Automated Insulin Delivery Systems. Diabetes Technol. Ther..

[2] Hojbjerre L., Skov-Jensen C., Kaastrup P., Pedersen P.E., Stallknecht B. (2009). Effect of steel and teflon infusion catheters on subcutaneous adipose tissue blood flow and infusion counter pressure in humans. Diabetes Technol. Ther..

[3] Patel P.J., Benasi K., Ferrari G., Evans M.G., Shanmugham S., Wilson D.M., Buckingham B.A. (2014). Randomized trial of infusion set function: Steel versus teflon. Diabetes Technol. Ther..

[4] Hauzenberger J.R., Munzker J., Kotzbeck P., Asslaber M., Bubalo V., Joseph J.I., Pieber T.R. (2018). Systematic in vivo evaluation of the time-dependent inflammatory response to steel and Teflon insulin infusion catheters. Sci. Rep..

[5] Teska B.M., Alarcon J., Pettis R.J., Randolph T.W., Carpenter J.F. (2014). Effects of phenol and meta-cresol depletion on insulin analog stability at physiological temperature. J. Pharm. Sci..

[6] Lewis B.E., Mulka A., Mao L., Sharafieh R., Qiao Y., Kesserwan S., Wu R., Kreutzer D., Klueh U. (2021). Insulin Derived Fibrils Induce Cytotoxicity in vitro and Trigger Inflammation in Murine Models. J. Diabetes Sci. Technol..

[7] Kesserwan S., Mulka A., Sharafieh R., Qiao Y., Wu R., Kreutzer D.L., Klueh U. (2021). Advancing continuous subcutaneous insulin infusion in vivo: New insights into tissue challenges. J. Biomed. Mater. Res. A.

[8] Mulka A., Lewis B.E., Mao L., Sharafieh R., Kesserwan S., Wu R., Kreutzer D.L., Klueh U. (2021). Phenolic Preservative Removal from Commercial Insulin Formulations Reduces Tissue Inflammation while Maintaining Euglycemia. ACS Pharmacol. Transl. Sci..

[9] Weber C., Kammerer D., Streit B., Licht A.H. (2015). Phenolic excipients of insulin formulations induce cell death, pro-inflammatory signaling and MCP-1 release. Toxicol. Rep..

[10] Kesserwan S., Lewis B.E., Mao L., Sharafieh R., Atwood T., Kreutzer D.L., Klueh U. (2022). Inflammation at Site of Insulin Infusion Diminishes Glycemic Control. J. Pharm. Sci..

[11] Kesserwan S., Mao L., Sharafieh R., Kreutzer D.L., Klueh U. (2022). A pharmacological approach assessing the role of mast cells in insulin infusion site inflammation. Drug Deliv. Transl. Res..

[12] Arumugam T., Ramachandran V., Logsdon C.D. (2006). Effect of cromolyn on S100P interactions with RAGE and pancreatic cancer growth and invasion in mouse models. J. Natl. Cancer Inst..

[13] Galli S.J., Tsai M., Marichal T., Tchougounova E., Reber L.L., Pejler G. (2015). Approaches for analyzing the roles of mast cells and their proteases in vivo. Adv. Immunol..

[14] Wilgus T.A., Ud-Din S., Bayat A. (2020). A Review of the Evidence for and against a Role for Mast Cells in Cutaneous Scarring and Fibrosis. Int. J. Mol. Sci..

[15] Otsuka A., Nonomura Y., Kabashima K. (2016). Roles of basophils and mast cells in cutaneous inflammation. Semin. Immunopathol..

[16] Benyon R.C. (1989). The human skin mast cell. Clin. Exp. Allergy.

[17] Komi D.E.A., Khomtchouk K., Santa Maria P.L. (2020). A Review of the Contribution of Mast Cells in Wound Healing: Involved Molecular and Cellular Mechanisms. Clin. Rev. Allergy Immunol..

[18] Grimbaldeston M.A., Chen C.C., Piliponsky A.M., Tsai M., Tam S.Y., Galli S.J. (2005). Mast cell-deficient *W-sash c-kit* mutant *Kit W-sh/W-sh* mice as a model for investigating mast cell biology in vivo. Am. J. Pathol..

[19] Reber L.L., Marichal T., Galli S.J. (2012). New models for analyzing mast cell functions in vivo. Trends Immunol..

[20] Nagle D.L., Kozak C.A., Mano H., Chapman V.M., Bucan M. (1995). Physical mapping of the *Tec* and *Gabrb1 loci* reveals that the Wsh mutation on mouse chromosome 5 is associated with an inversion. Hum. Mol. Genet..

[21] Dahlin J.S., Maurer M., Metcalfe D.D., Pejler G., Sagi-Eisenberg R., Nilsson G. (2022). The ingenious mast cell: Contemporary insights into mast cell behavior and function. Allergy.

[22] Nigrovic P.A., Gray D.H., Jones T., Hallgren J., Kuo F.C., Chaletzky B., Gurish M., Mathis D., Benoist C., Lee D.M. (2008). Genetic inversion in mast cell-deficient (*Wsh*) mice interrupts corin and manifests as hematopoietic and cardiac aberrancy. Am. J. Pathol..

[23] Piliponsky A.M., Chen C.C., Grimbaldeston M.A., Burns-Guydish S.M., Hardy J., Kalesnikoff J., Contag C.H., Tsai M., Galli S.J. (2010). Mast cell-derived TNF can exacerbate mortality during severe bacterial infections in C57BL/6-KitW-sh/W-sh mice. Am. J. Pathol..

[24] Wu K.K., Huan Y. (2008). Streptozotocin-induced diabetic models in mice and rats. Curr. Protoc. Pharmacol..

[25] Lilla J.N., Chen C.C., Mukai K., BenBarak M.J., Franco C.B., Kalesnikoff J., Yu M., Tsai M., Piliponsky A.M., Galli S.J. (2011). Reduced mast cell and basophil numbers and function in *Cpa3-Cre*; *Mcl-1fl/fl* mice. Blood.

[26] Ribatti D. (2018). The Staining of Mast Cells: A Historical Overview. Int. Arch. Allergy Immunol..

[27] Dong J., Chen L., Zhang Y., Jayaswal N., Mezghani I., Zhang W., Veves A. (2020). Mast Cells in Diabetes and Diabetic Wound Healing. Adv. Ther..

[28] Tellechea A., Leal E.C., Kafanas A., Auster M.E., Kuchibhotla S., Ostrovsky Y., Tecilazich F., Baltzis D., Zheng Y., Carvalho E. (2016). Mast Cells Regulate Wound Healing in Diabetes. Diabetes.

[29] Klueh U., Kaur M., Qiao Y., Kreutzer D.L. (2010). Critical role of tissue mast cells in controlling long-term glucose sensor function in vivo. Biomaterials.

[30] Orenstein S.B., Saberski E.R., Klueh U., Kreutzer D.L., Novitsky Y.W. (2010). Effects of mast cell modulation on early host response to implanted synthetic meshes. Hernia J. Hernias Abdom. Wall Surg..

[31] Maurer M., Taube C., Schröder N.W.J., Ebmeyer J., Siebenhaar F., Geldmacher A., Schubert N., Roers A. (2019). Mast cells drive IgE-mediated disease but might be bystanders in many other inflammatory and neoplastic conditions. J. Allergy Clin. Immunol..

[32] Luo Y., Meyer N., Jiao Q., Scheffel J., Zimmermann C., Metz M., Zenclussen A., Maurer M., Siebenhaar F. (2019). *Chymase-Cre*; *Mcl-1(fl/fl*) Mice Exhibit Reduced Numbers of Mucosal Mast Cells. Front. Immunol..

[33] Peschke K., Dudeck A., Rabenhorst A., Hartmann K., Roers A. (2015). *Cre/loxP*-based mouse models of mast cell deficiency and mast cell-specific gene inactivation. Methods Mol. Biol..

[34] De Filippo K., Dudeck A., Hasenberg M., Nye E., van Rooijen N., Hartmann K., Gunzer M., Roers A., Hogg N. (2013). Mast cell and macrophage chemokines CXCL1/CXCL2 control the early stage of neutrophil recruitment during tissue inflammation. Blood.

[35] Lopes J.P., Stylianou M., Backman E., Holmberg S., Ekoff M., Nilsson G., Urban C.F. (2019). Cryptococcus neoformans Induces MCP-1 Release and Delays the Death of Human Mast Cells. Front. Cell Infect. Microbiol..

[36] Rodriguez A.R., Yu J.-J., Navara C., Chambers J.P., Guentzel M.N., Arulanandam B.P. (2016). Contribution of FcɛRI-associated vesicles to mast cell–macrophage communication following Francisella tularensis infection. Innate Immun..

[37] Amini A., Soleimani H., Rezaei F., Ghoreishi S.K., Chien S., Bayat M. (2021). The Combined Effect of Photobiomodulation and Curcumin on Acute Skin Wound Healing in Rats. J. Lasers Med. Sci..

[38] Delamaire M., Maugendre D., Moreno M., Le Goff M.C., Allannic H., Genetet B. (1997). Impaired leucocyte functions in diabetic patients. Diabet. Med..

[39] Kumar M., Roe K., Nerurkar P.V., Orillo B., Thompson K.S., Verma S., Nerurkar V.R. (2014). Reduced immune cell infiltration and increased pro-inflammatory mediators in the brain of Type 2 diabetic mouse model infected with West Nile virus. J. Neuroinflammat..

